# m6A-related lncRNAs are potential biomarkers for the prognosis of COAD patients

**DOI:** 10.3389/fonc.2022.920023

**Published:** 2022-08-30

**Authors:** Chenyang Xu, Tingting He, Xinxin Shao, Ling Gao, Lei Cao

**Affiliations:** ^1^ Department of General Surgery, The First Affiliated Hospital of Soochow University, Suzhou, China; ^2^ Department of Interventional Radiology, The First Affiliated Hospital of Soochow University, Suzhou, China; ^3^ Jiangsu Key Laboratory of Clinical Immunology, Jiangsu Institute of Clinical Immunology, The First Affiliated Hospital of Soochow University, Suzhou, China

**Keywords:** m6A modification, colon adenocarcinoma, lncRNA, cell function assays, biomarker

## Abstract

**Background:**

Colon adenocarcinoma (COAD) is the most common subtype of colon cancer. However, the 5-year survival rate of COAD patients remains unsatisfactory. N6-methyladenosine (m6A) and long noncoding RNAs (lncRNAs) play essential roles in the occurrence and development of COAD. Herein, we are committed to establish and validate a prognostic m6A-related lncRNA signature.

**Methods:**

We obtained m6A-related lncRNAs by coexpression. The m6A-related lncRNA risk signature (m6ALncSig) was developed *via* univariate, LASSO, and multivariate Cox regression analyses. Kaplan-Meier (KM) survival curves, gene set enrichment analysis (GSEA), and nomogram generation were conducted to assess m6ALncSig. In addition, the potential immunotherapeutic signatures were also discussed. Real-time PCR and CCK8 analysis were performed to evaluate the expression and functions of lncRNA UBA6-AS1, which was selected.

**Results:**

The risk signature comprising 14 m6A-related lncRNAs (m6ALncSig) was established, which possessed a superior predictive ability of prognosis. Meanwhile, m6ALncSig was linked to immune cell infiltration. The level of UBA6-AS1 expression was validated in 17 pairs of COAD samples. In cell function experiments, UBA6-AS1 knockdown attenuated cell proliferation capacity.

**Conclusions:**

Collectively, m6ALncSig could serve as an independent predictive factor for COAD and accurately estimate the outcome for COAD patients. Importantly, UBA6-AS1 was first identified as an oncogene in COAD.

## Introduction

Colon adenocarcinoma (COAD) is the most frequent sub-type of colon cancer (1). With the development of diagnostic methods and comprehensive treatment recently, the clinical prognosis of patients with COAD has dramatically improved. Nonetheless, the 5-year survival rate for patients with COAD remains unsatisfactory (2). Currently, numerous investigations show that the identification and utility of molecular markers can offer tremendous clinical value for cancer therapy (3).

As the most common RNA modification, N6-methyladenosine (m6A) plays a critical role in various biological processes (4). The m6A RNA modification is reversible and dynamically regulated by methyltransferases (writers), m6A-binding proteins (readers), and demethylases (erasers) (5, 6). Various cell functions are influenced by the chemical structure of RNA (7). Thus, long noncoding RNAs (lncRNAs) regulated by m6A modification may play crucial role in oncogenesis and cancer development.

Recent research has demonstrated that m6A modification is closely linked to tumor progression. For example, METTL14 can suppress growth and metastasis of renal cell carcinoma by reducing lncRNA NEAT1 (8). Besides, m6A-mediated up-regulation of lncRNA LIFR-AS1 enhances the progression of pancreatic cancer *via* miRNA-150-5p/VEGFA/Akt signaling (9). Recently, another investigation has suggested that dysregulated m6A modification is tightly associated with COAD (10). However, the potential functions of lncRNA m6A methylation remain unclear. Hence, comprehensive understanding of m6A-related lncRNAs may be of great clinical value for COAD patients.

Herein, we extracted the expression matrixes of 24 m6A modulators and 14142 lncRNAs from the TCGA cohort. Then, Pearson correlation analysis was adopted to identify the m6A-related lncRNAs. The m6A-related lncRNA signature (m6ALncSig) was developed to estimate the overall survival (OS) of patients with COAD. Moreover, we investigated the correlation between immunotherapy responses and m6ALncSig. Finally, a nomogram was generated to estimate the probability of 1-, 3-, and 5-year OS.

## Materials and methods

### Processing of data sets

**Figure 1:**
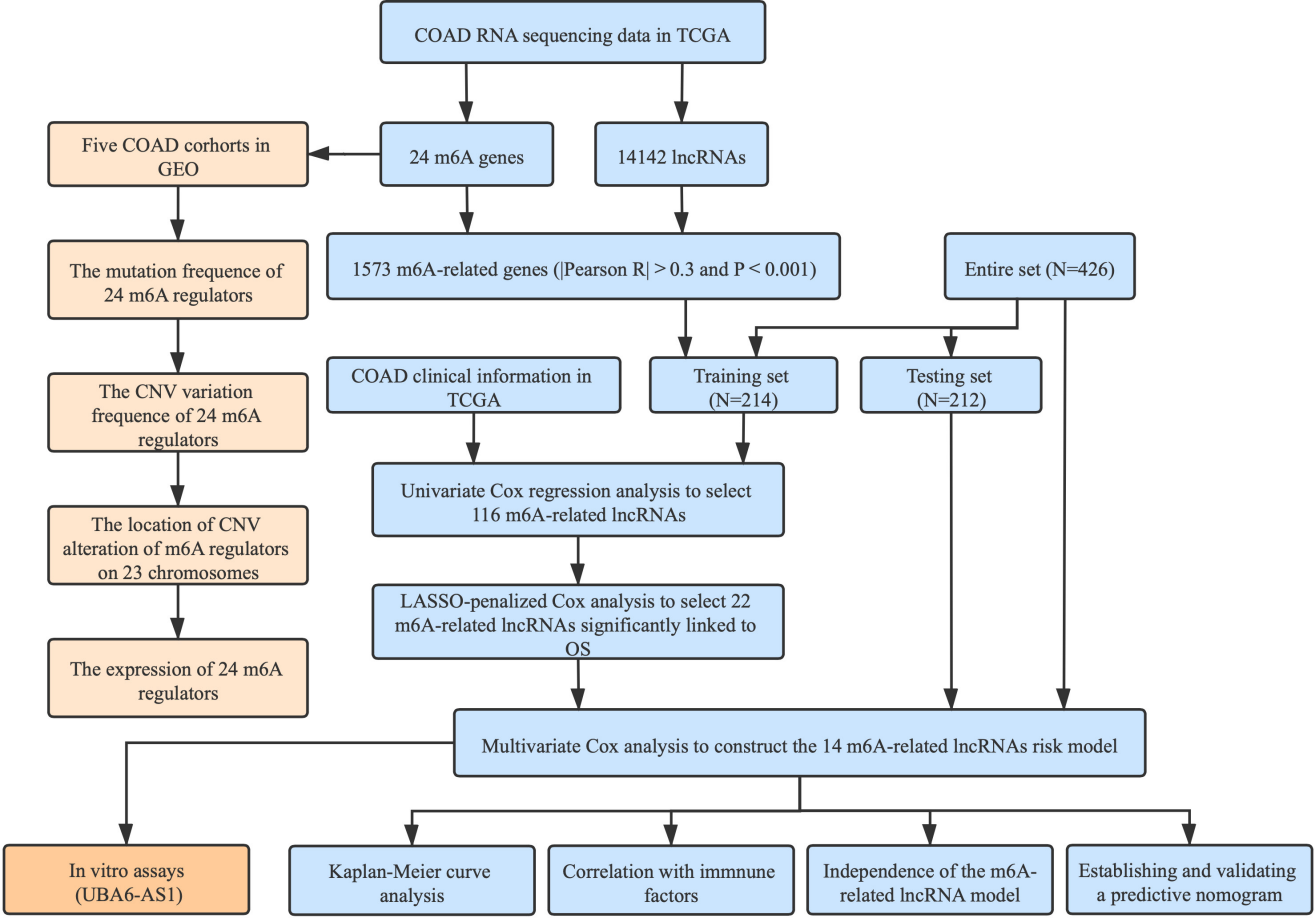
Flowchart of this study.

The detailed workflow for this research is given in **Figure 1**. We downloaded RNA sequencing data, corresponding clinical information along with mutation data from the TCGA database with VarScan software. In order to reduce statistical bias, COAD patients with missing OS values were excluded. Additionally, six eligible colon cancer cohorts (GSE39582, GSE38832, GSE37892, GSE33113, GSE29621, and GSE17536) were obtained from the GEO database for further research.

### Identification of m6A-related lncRNAs

Pearson correlation analysis was carried out to screen m6A-related lncRNAs, and 1573 m6A-related lncRNAs were identified with the criteria of |Pearson R| > 0.3 and p < 0.001 (11, 12).

### Establishment and validation of m6ALncSig

By means of createDataPartition function, we divided the entire TCGA set randomly into the training set and the testing set based on survival status. Meanwhile, mortality was ensured to be consistent between two sets. The baseline characteristics of the two sets were presented in **Table S1**. We employed the training dataset to develop an m6ALncSig, and the testing set and entire set served as the validation sets. Univariate Cox regression was utilized to screen the prognostic m6A-related lncRNAs. To avoid overfitting, LASSO regression was introduced *via* the glmnet R package (10-fold cross-validation). Ultimately, we applied multivariate regression to establish the m6ALncSig. The clinical characteristics were transformed into dichotomous variables, including sex, risk score, TNM stage, age, and tumor grade.

### Functional analysis and KM survival analysis

Gene set enrichment was analyzed with GSEA. To evaluate survival differences between the high- and low-risk groups, we conducted KM survival analysis with the R packages survminer and survival.

### Exploration of m6ALncSig in immunotherapy

Based on the existing immune gene set, enrichment score for each immune component was quantified by the single sample Gene Set Enrichment Analysis (ssGSEA). We explored the somatic mutation data of patients with COAD *via* the R package Maftool. The tumor mutation burden (TMB) was calculated with tumor-specific mutation genes. Also, we applied the Tracking of Indels by DEcomposition (TIDE) algorithm to predict the immunotherapy response for each COAD patients.

### Independent analysis of m6AlncSig

To assess whether m6ALncSig was an independent predictive factor when combined with other clinical features, we conducted univariate along with multivariate Cox regression analyses.

### Establishment and validation of a predictive nomogram

A nomogram was constructed based on all prognostic factors (age, gender, TNM stage, and risk score) to predict the probability of 1-, 3-, and 5-year OS. Afterward, we plotted calibration curves to assess the predictive capacity of the nomogram. The adjustment factors included age, gender, stage, and TNM stage.

### *In vitro* assays

The normal and tumor tissues were acquired from COAD patients who had been treated with surgery at the First Affiliated Hospital of Soochow University. Our research work was also authorized by the Ethics Committee at the First Affiliated Hospital of Soochow University. The cell lines RKO, HCT116, NCM460, and SW620 were purchased from ATCC and cultured in DMEM (Gibco, USA) supplemented with 10% FBS (Gibco, USA) and 1% penicillin–streptomycin. The siRNA targeting UBA6-AS1 was designed and produced by GenePharma (Suzhou, China). The sequences of siRNAs were given in **Table S2**.

To detect the expression of m6A-related lncRNAs, quantitative real-time PCR was performed after RNA extraction and reverse transcription. The primer sequences were shown in **Table S2**. The transfected cells were made into cell suspensions and cultured in 96 well plates (3000/well). The original medium was removed and replaced by serum-free medium containing 10 µl CCK8 reagent (NCM Biotech, China). After 2 h of incubation, values of OD 450 nm were measured by Multiskan FC (Thermo Fisher Scientific).

### Statistical analysis

All statistical analyses were implemented in R software. KM survival analysis was carried out *via* Log-Rank test. Each experiment was performed in triplicate and repeated three times. One-way analysis of variance (ANOVA) or the Student’s t-test was utilized to perform statistical analyses, with P < 0.05 signifying statistical significance.

## Results

### The landscape of m6A RNA methylation regulators in COAD

**Figure 2:**
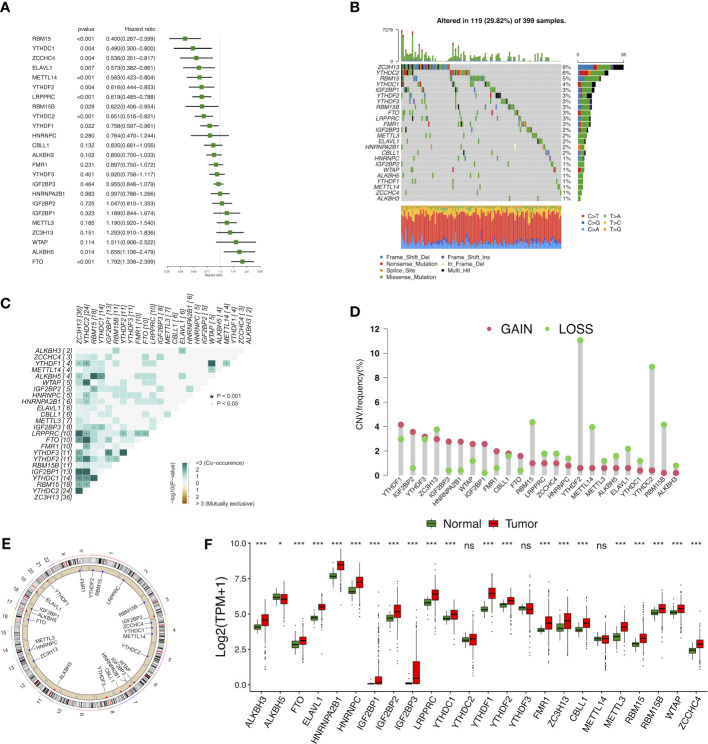
Landscape of genetic and expression variation of m6A modulators in colon cancer. **(A)** The prognostic analyses for 24 m6A modulators in the five colon cancer cohorts with univariate Cox regression. **(B)** The mutation frequency of 24 m6A modulators. **(C)** The mutation co-occurrence along with exclusion analyses for 24 m6A modulators. **(D)** The CNV variation frequency of 24 m6A modulators. **(E)** The location of CNV alteration of m6A modulators on 23 chromosomes. **(F)** The expression of 24 m6A modulators between normal tissues and tumor tissues. ns, Not significant; *p < 0.05, **p < 0.01, ***p < 0.001.

In total, 24 m6A modulators were identified for subsequent analysis. A univariate Cox regression model demonstrated the prognostic values of 24 m6A modulators in COAD patients (**Figure 2A**). Among 399 samples, 119 exhibited m6A modulator mutations, with a frequency of 29.82%. All 24 m6A modulators experienced the mutations in COAD patients, with ZC3H13 harboring the greatest mutation frequency followed by YTHDC2 **(** **Figure 2B** **)**. Further analysis showed the significant co-occurrence relationship between the majorities of 24 m6A regulators (**Figure 2C**). The assessment of copy number variation (CNV) alteration frequency revealed a widespread CNV variation in 24 m6A regulators, whereas YTHDF2, YTHDC2, RBM15, RBM15B, and METTL14 exhibited more copy number deletions (**Figure 2D**). The location of CNV alteration of m6A modulators on chromosomes was shown in **Figure 2E**. To determine whether the CNV variations affected the expression of m6A modulators in COAD patients, we evaluated the mRNA expression levels of modulators between cancerous and non-cancerous samples. Compared to normal colon tissues, a great number of m6A regulators with CNV deletions had lower expression in colon cancer tissues (for instance, ALKBH5), and vice versa (e.g., YTHDF1, IGF2BP2, HNRNPA2B1, etc.) (**Figure 2F**). However, not all the regulators were in accordance with above conclusion, as gene expression was regulated not only by CNV but also by DNA methylation and transcription factors. Overall, our results indicated that m6A modulators played an indispensable role in colon cancer oncogenesis and progression.

### Identification of m6A-related lncRNAs in patients with COAD

**Figure 3:**
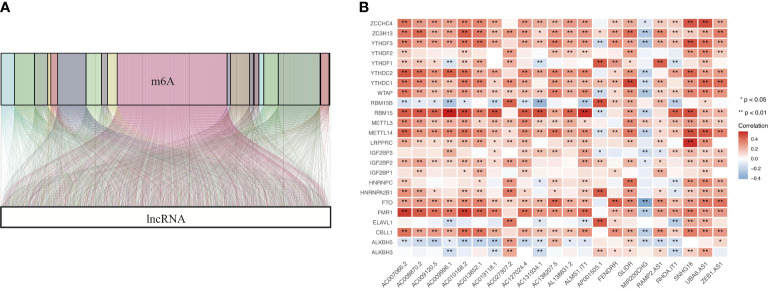
Identification of m6A-related lncRNAs in COAD. **(A)** Sankey coexpression diagram for m6A modulators and m6A-related lncRNAs. **(B)** Heatmap exhibiting the correlations of 24 m6A modulators with 22 prognostic m6A-related lncRNAs.

We extracted the expression matrixes of 24 m6A modulators and 14142 lncRNAs from the TCGA cohort. Next, we utilized a Sankey diagram to visualize the m6A-lncRNA co-expression relationship, and 1573 lncRNAs was identified as m6A-related lncRNAs (|Pearson R| > 0.3 and p < 0.001) (**Figure 3A**). Finally, the correlation heat map summarized the significant correlations of m6A modulators with lncRNAs in the TCGA entire set (**Figure 3B**).

### Cluster analysis of m6A-related lncRNAs

**Figure 4:**
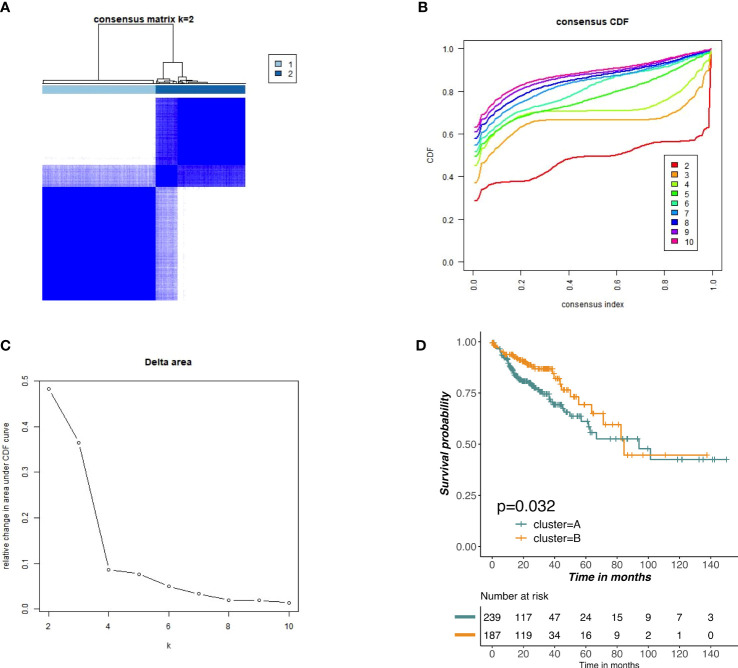
Unsupervised clustering of prognostic m6A-related lncRNAs. **(A–C)** Consensus clustering identified two main tumors clusters based on the expression of m6A-related lncRNA. **(D)** KM curves of OS for two clusters in COAD.

Based on the expression profiles of m6A-related lncRNAs, we performed unsupervised clustering to partition colon tumor samples into different subgroups and k = 2 was attained as the optimal clustering parameter (**Figures 4A–C**). To further investigate whether there was a survival difference in two subgroups, KM survival curve was performed for overall survival. The results indicated that cluster B had significantly better survival than cluster A (**Figure 4D**).

### Construction and verification of m6ALncSig in COAD patients

**Figure 5:**
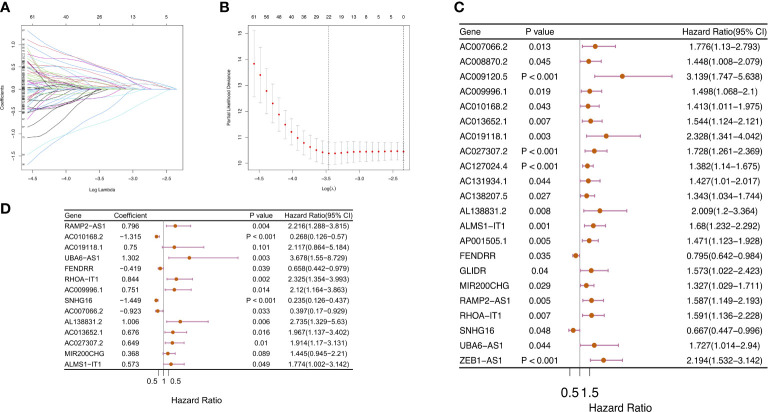
Construction of m6ALncSig for patients with COAD. **(A–C)** LASSO-penalized COX regression analysis selected 22 m6A-related lncRNA remarkably associated with OS. **(D)** Multivariate Cox regression analysis revealed 14 independent prognostic lncRNAs.

**Figure 6:**
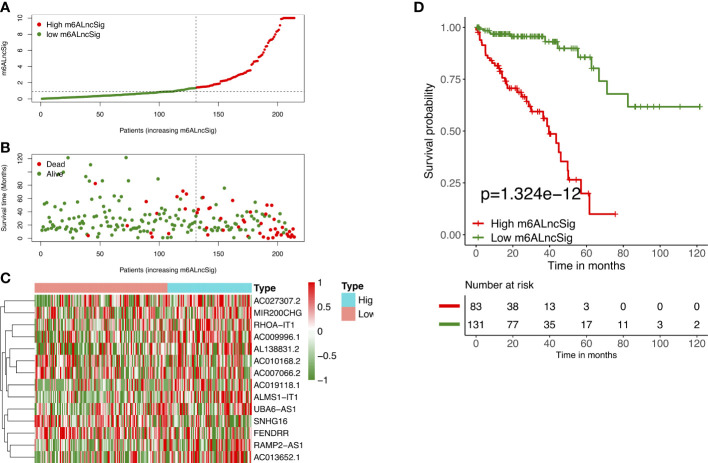
Prognostic value of m6ALncSig in the TCGA training set. **(A)** Distribution of m6ALncSig-based risk score. **(B)** Survival status along with survival time of COAD patients between high- and low-risk groups. **(C)** Expression standards of the 14 prognostic m6A-related lncRNAs. **(D)** KM curves of OS in the high- and low-risk groups.

We employed univariate Cox regression along with LASSO regression to determine 22 prognostic m6A-related lncRNAs (**Figures 5A–C**). Next, multivariate analysis was performed to identify independent prognostic factors in the training set. Finally, 14 prognostic lncRNAs were chosen to develop an m6ALncSig (**Figure 5D**). We divided COAD samples into high- and low-risk groups on the basis of the median risk score (**Figure 6A**) and assess risk scores of each sample. It was found that COAD patients in the low-risk group exhibited better survival status than those in the high-risk group (**Figure 6B**). The relative expression levels of 14 m6A-related lncRNAs were presented in **Figure 6C**. Besides, the survival curve revealed that the high-risk group had a lower survival rate in comparison with the low-risk group (p =1.32e-12) (**Figure 6D**).

**Figure 7:**
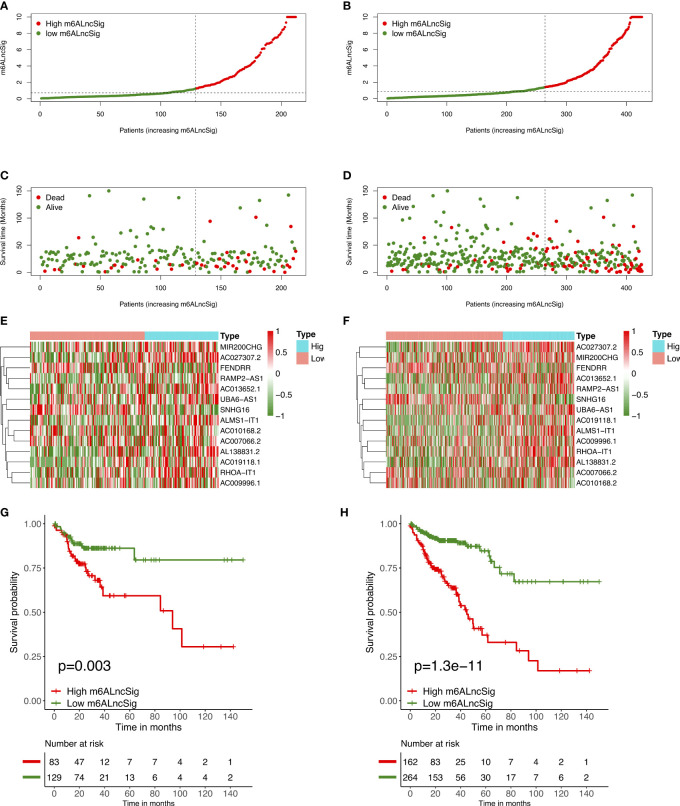
Prognostic value of m6ALncSig in the TCGA testing and entire sets. **(A)** Distribution of m6ALncSig-based risk score for the testing set. **(B)** Survival status along with survival time of COAD patients between high- and low-risk groups for the testing set. **(C)** Expression standards of the 14 prognostic m6A-related lncRNAs for the testing set. **(D)** Kaplan–Meier curves of OS in the high- and low-risk groups for the testing set. **(E)** Distribution of m6ALncSig-based risk score for the entire set. **(F)** Survival status and survival time of patients with COAD between high- and low-risk groups for the entire set. **(G)** Expression standards of the 14 prognostic m6A-related lncRNAs for the entire set. **(H)** KM curves of OS in the high- and low-risk groups for the entire set.

**Figure 8:**
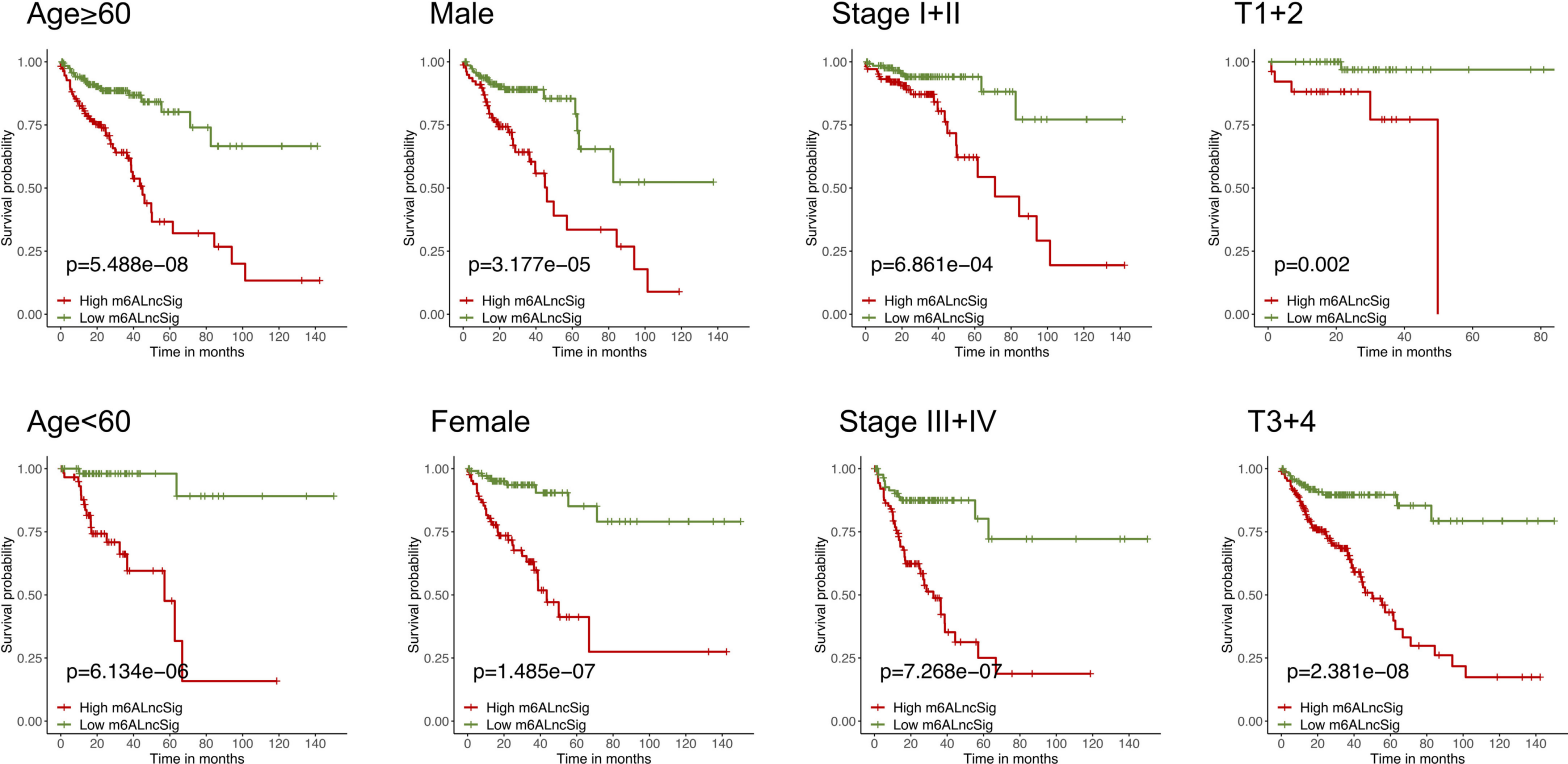
KM curves stratified by age, gender, tumor grade, and TNM stage between the high- and low-risk groups in the TCGA entire set.

To verify the prognostic capability of m6ALncSig, we assessed risk scores of each COAD sample in the test set and entire set. The results were in good agreement with those obtained in the training set (**Figures 7A–F**). Meanwhile, KM survival analysis performed on the testing set and entire set exhibited no difference with the outcomes in the training set, illustrating that COAD patients in the low-risk group had a higher survival rate than the high-risk group (**Figures 7G, H** **)**. To further validate the ability of m6ALncSig, DSS, PFI, and DFI were explored to observe the difference between high- and low-risk groups **(** **Figures S1A–C** **).** As expected, the high-risk group had a worse prognosis, indicating that m6ALncSig could accurately predict prognosis of COAD patients. Besides, on the basis of clinical stratification analysis based on age, gender, stage, and tumor stage, the OS of the low-risk group was found to be superior to that of the high-risk group (**Figure 8**).

### Estimation of the performance of m6ALncSig in the tumor microenvironment and immunotherapy response

**Figure 9:**
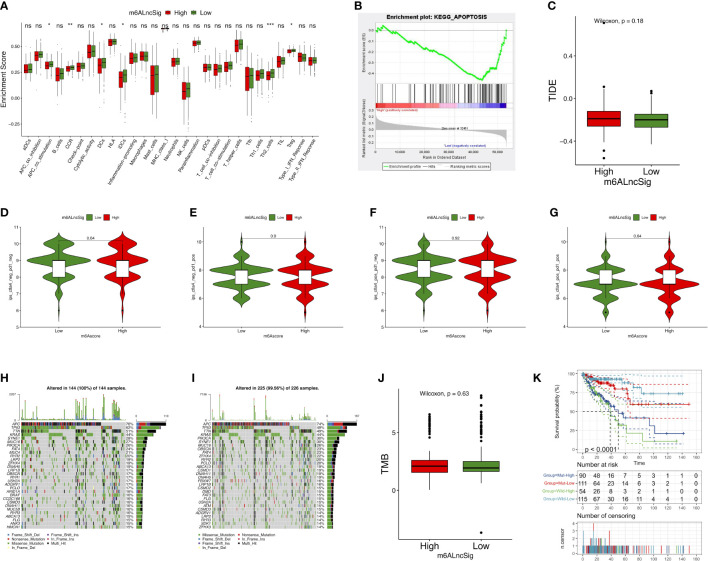
Evaluation of tumor immune microenvironment (TIME) and tumor immunotherapy response using m6ALncSig in the TCGA entire set. **(A)** The box diagram illustrating the different infiltration levels of immune cells in the high- and low-risk groups. **(B)** Gene Sets Enrichment Analysis (GSEA). **(C)** The predicted difference of TIDE in the high- and low-risk groups. **(D–G)** The correlation of the risk scores with IPS in four subgroups, CTLA4^−^ PD1^−^ **(D)**, CTLA4^−^ PD1^+^ **(E)**, CTLA4^+^ PD1^−^ **(F)**, and CTLA4^+^ PD1^+^ **(F)**. **(H, I)** Waterfall plot of the genes with high mutation frequency in the high- **(H)** and low-risk groups **(I)**. **(J)** TMB difference between the high- and low-risk groups. **(K)** KM curve for patients classified based on TP53 mutation status and m6ALncSig. ns, Not significant; *p < 0.05, **p < 0.01, ***p < 0.001.

The enrichment level of immune functions and pathways in COAD were analyzed based on m6ALncSig. The expression levels of several immune indicators showed a significant difference between high- and low-risk groups, such as Th2 cells, Treg cells, and APC co-stimulation (**Figure 9A**). GSEA results revealed that the survival difference between high- and low-risk groups of m6ALncSig could be caused by the apoptosis pathway activation (**Figure 9B**). We next examined the correlation between m6ALncSig and immunotherapeutic biomarkers. The response to immunotherapy had no difference between high- and low-risk groups (**Figures 9C–G**). Furthermore, the top 30 driver genes with the highest mutant frequency between high- and low-risk groups were shown in **Figures 9H, I**. According to TCGA somatic mutation data, we calculated TMB scores. Similarly, the TMB had no difference between two groups (**Figure 9J**). In summary, m6ALncSig could not accurately predict immunotherapy efficacy. However, we found that m6ALncSig might have better predictive abilities than the TP53 mutation status. As shown in **Figure 9K**, the patients with TP53 mutation (wild/mutation) in the high-risk group showed similar survival, indicating that TP53 mutation were unable to distinguish the survival rate of the high-risk group. More interestingly, compared to patients with TP53 mutation in the low-risk group, patients with wild-type TP53 in the high-risk group had a worse prognosis (**Figure 9K**). Thus, m6ALncSig showed more powerful prognostic significance than TP53 mutation status.

### Evaluation of m6ALncSig and clinical features of COAD

**Figure 10:**
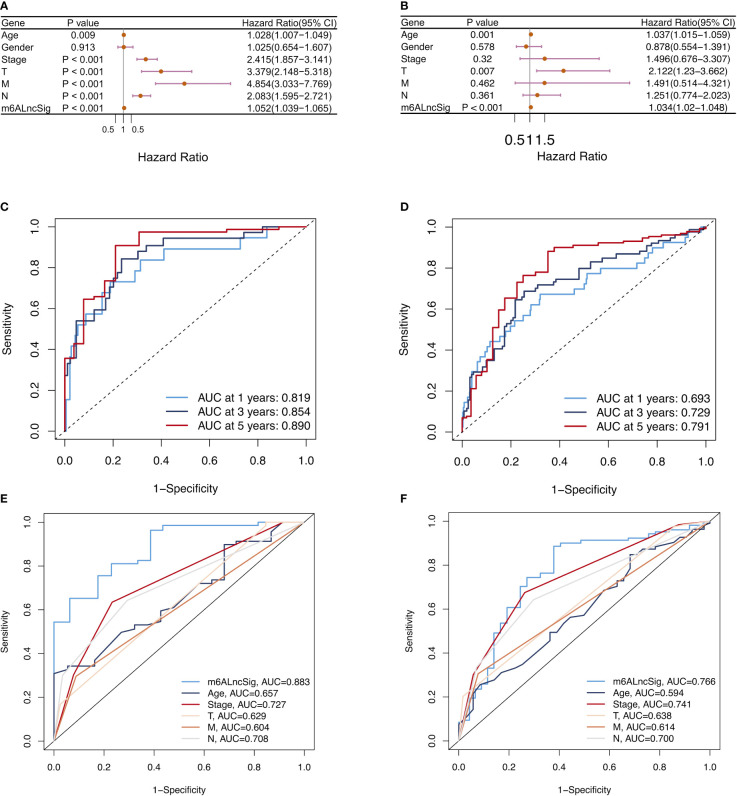
Assessment of m6ALncSig and clinicopathological features in the TCGA training and entire sets. **(A, B)** Univariate and multivariate Cox regression analysis of clinicopathological features and risk score. **(C, D)** ROC curves of the training and entire sets at 1, 3, and 5 years. **(E, F)** ROC curves for the clinical characteristics and risk score in the training and entire sets.

To assess whether the m6ALncSig was an independent prognostic indicator, we performed univariate and multivariate Cox regression analyses to compare the prognostic values of risk score with other clinical characteristics. Univariate Cox analysis indicated that signature-based risk score was significantly associated with prognosis and independent of other clinical features (HR: 1.052, 95% CI: 1.039–1.065, p < 0.001; **Figure 10A**). Simultaneously, multivariate Cox analysis further demonstrated the independence of the signature (HR: 1.034, 95% CI: 1.020–1.048, p < 0.001; **Figure 10B**). Afterwards, the ROC curves were performed to assess the accuracy of m6ALncSig in predicting survival of COAD patients at 1, 3, and 5 years (Training set: 1-year AUC = 0.819, 3-year AUC = 0.854, 5-year AUC = 0.890; All set: 1-year AUC = 0.693, 3-year AUC = 0.729, 5-year AUC = 0.791; **Figures 10C, D** **)**. In addition, the AUCs of the risk grade in the training set and all set were higher than the AUCs of other clinicopathologic features, suggesting that m6ALncSig was extremely reliable in predicting prognosis (**Figures 10E, F** **)**.

### Development and validation of the prognostic nomogram

**Figure 11:**
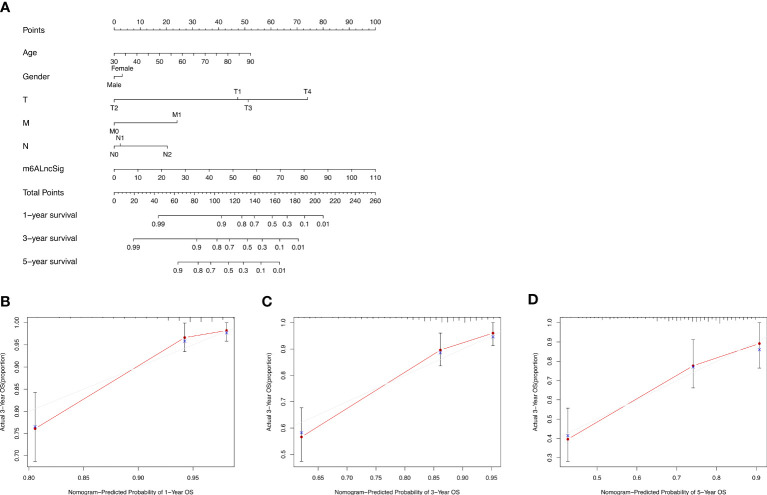
Nomogram and performance evaluation. **(A)** Nomogram predicting 1-, 3- and 5-year OS for patients with COAD. **(B–D)** Calibration plot of the nomogram for 1-, 3- and 5-year OS.

Based on the survival analysis, the nomogram comprising the risk score and other clinicopathological characteristics was developed to predict 1-, 3-, or 5-year OS. In comparison to clinicopathological factors, the risk score of the m6ALncSig demonstrated more prominent predictive power in the nomogram (**Figure 11A**). Concurrently, the calibration curve revealed good agreement among the estimations with the nomogram and actual outcomes (**Figures 11B–D**).

### *In vitro* assays

**Figure 12:**
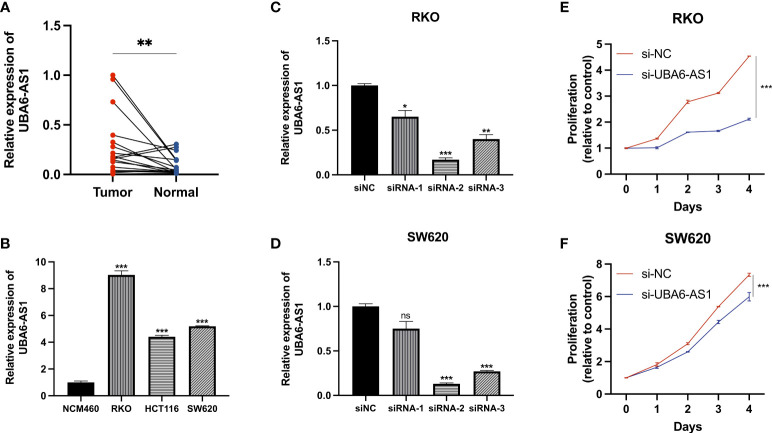
Cell function assays. **(A, B)** Real-time PCR. **(C, D)** The transfection efficiencies of three siRNAs targeting UBA6-AS1 in RKO cells **(C)** and SW620 cells **(D)**. **(E, F)** CCK-8 assays were conducted after UBA6-AS1 knockdown in RKO **(E)** and SW620 **(F)**. ns, Not significant; *p < 0.05, **p < 0.01, ***p < 0.001.

LncRNA UBA6-AS1 was significantly linked to the greatest number of m6A regulators (**Figure 3B**) and showed the highest hazard ratio (**Figure 5D**). In addition, the expression level of UBA6-AS1 was significantly upregulated in COAD tumor tissues (**Supplementary Figure S2A**). Patients with low UBA6-AS1 expression had better survival than those with high UBA6-AS1 expression **(** **Supplementary Figure S2B** **)**. The AUC was 0.661, suggesting that UBA6-AS1 could be served as an ideal biomarker to distinguish tumor from non-tumor tissues **(** **Supplementary Figure S2C** **)**. Besides, the results of logistic regression analysis suggested that UBA6-AS1 was significantly associated age, N stage and pathologic stage (**Supplementary Figures S2D–F**). According to the analysis, we speculated that UBA6-AS1 may play important roles in the occurrence and development of COAD. Therefore, we picked it as the candidate gene to perform the subsequent experiments. Real-time PCR results revealed that UBA6-AS1 was significantly upregulated in cancer tissues ( **Figure 12A** ) and cell lines ( **Figure 12B** ). The interference efficiencies of three siRNAs targeting UBA6-AS1 were detected, which demonstrated that siRNA-2 exhibited the best interference efficiency in RKO and SW620 cells **(** **Figures 12C, D** **)**. CCK-8 assay revealed a significant reduction in cell viability after siRNA interference (**Figures 12E, F**).

## Discussion

COAD is a common malignant tumor with high mortality (13). Recently, there are increasing numbers of studies that focus on exploring the onset and progress of COAD. Current studies have indicated that the difference of colon cancer subtypes can lead to distinct tumor characteristics and clinical outcomes (14). Thus, it is necessary to identify signatures with lncRNAs for the survival prediction of COAD patients.

As the most common RNA modification, m6A not only affects mRNA metabolism but also appears to be involved with the regulation of noncoding RNA (15–17). Currently, studies about lncRNA have drawn much attention in various cancer fields. Many lncRNAs can be modified by specific m6A modulators to participate in the tumorigenesis and development (18–20). Studies have documented that lncRNAs can serve as competitive endogenous RNAs to target m6A modulators, influencing vital cellular functions (21). Additionally, m6A modification can maintains the stabilization of lncRNAs by changing local RNA structure (22). Both lncRNAs and m6A modification are key factors in tumor occurrence and development. However, there are few studies on the predictive markers of COAD regarding m6A-related lncRNAs. Consequently, we attempted to generate an m6A-related lncRNA risk signature.

Herein, 1573 m6A-related lncRNAs were screened from the TCGA dataset for exploring the prognostic value of m6A-related lncRNAs. We finally construct a 14-gene signature (m6ALncSig) to predict OS of COAD. Among all of them, FENDRR inhibits colorectal cancer progression by sponging miR-424-5p (23). Alternatively, as autophagy-related lncRNAs, SNHG16 and AC027307.2 can accurately predict the survival of COAD patients (24). Meanwhile, AC013652.1 and ALMS1-IT1 proved to be two ferroptosis-related lncRNAs associated with COAD prognosis (25). The other lncRNAs were first discovered in COAD. For example, lncRNA UBA6-AS1 was first shown to be highly expressed in COAD tumor tissues and regulate cell proliferation. We separated COAD samples into high- and low-risk groups on the basis of the median risk score of m6ALncSig. Obviously, the low-risk group exhibited better OS relative to the high-risk group. The m6ALncSig was identified as an independent factor by multivariate regression analysis. ROC analysis revealed that the m6ALncSig was superior to other clinicopathologic features in predicting prognosis for COAD patients. Additionally, we established a nomogram for predicting 1-, 3-, or 5-year OS. Not surprisingly, the calibration curves exhibited high concordance between the estimations of the nomogram and actual outcomes. In summary, as an independent prognostic factor of COAD, m6ALncSig can identify novel prognostic markers for further research.

TMB constitutes the total number of somatic mutations (26, 27). Recent studies exhibited that TMB can well estimate the response to PD-L1 treatment (28). Combined with TIDE algorithm, we found there were no significant differences between two risk groups in terms of immunotherapy response. Therefore, we infer that m6ALncSig may not have a capability to provide reliable biomarkers for tumor immunotherapy. According to the GSEA result, the most likely reason leading to this was the inhibition of tumor cell apoptosis in the high-risk group. The efficacy of immunotherapy is primarily dependent on the apoptosis of tumor cells (29) and overcoming apoptosis resistance is critical for the development of immune therapies (30). In addition, according to the ssGSEA result, the abundance of Th2 and Treg cells were significantly upregulated in the high-risk group. Th2 and Treg cells could induce immune tolerance, which is the main issues in cancer immunotherapy (31, 32).

In routine clinical practice, pathological stage is a key prognostic factor for COAD (33). However, the patients with the same cancer stage had different clinical outcomes, which indicated the present staging system was not sufficient for predicting prognosis (34). As such, novel prognostic markers need to be identified. Here, m6ALncSig provides a new approach to predict COAD prognosis and also gives important insights into the mechanism of lncRNA m6A modification.

In our study, we confirmed this novel signature in multiple ways. Nevertheless, there are several limitations in our study. First, m6ALncSig requires further external verification by more prospective clinical datasets. Alternatively, the biological mechanisms of m6A-related lncRNAs have not been completely elucidated. Thus, we should attempt to design more experiments for the exploration of functions and mechanisms.

## Conclusions

We screened 14 m6A-related lncRNAs significantly related to prognosis for establishing a predictive signature (m6ALncSig). Furthermore, m6ALncSig was capable of independently predicting the prognosis of COAD patients by combining molecular characteristics and clinical features. Moreover, UBA6-AS1 was first identified as an oncogene in colon cancer.

## Data availability statement

Publicly available datasets were analyzed in this study. This data can be found here: https://portal.gdc.cancer.gov/.

## Ethics statement

This study was reviewed and approved by The Ethics Committee of the First Affiliated Hospital of Soochow University. The patients/participants provided their written informed consent to participate in this study.

## Author contributions

CX conceived the work, conducted the bioinformatics analysis, and drafted the manuscript. TH collected the data and prepared the figures. XS helped designed the study. LG and LC were responsible for this work and reviewed the article critically. All authors contributed to the article and approved the submitted version.

## Funding

This work was funded by the National Natural Science Foundation of China (Grant No. 81974375).

## Conflict of interest

The authors declare that the research was conducted in the absence of any commercial or financial relationships that could be construed as a potential conflict of interest.

## Publisher’s note

All claims expressed in this article are solely those of the authors and do not necessarily represent those of their affiliated organizations, or those of the publisher, the editors and the reviewers. Any product that may be evaluated in this article, or claim that may be made by its manufacturer, is not guaranteed or endorsed by the publisher.

## Supplementary material

Supplementary Figure 1
**(A–C)** KM survival curves of DSS, PFI, and DFI between the high- and low-risk groups.

Supplementary Figure 2 The expression and prognosis of UBA6-AS1 in COAD. **(A)** The differential expression of UBA6-AS1 in COAD cancer and paracancer samples. **(B)** KM survival curve of UBA6-AS1. **(C)** ROC curve for UBA6-AS1. **(D)** Relationship between UBA6-AS1 expression with age, **(E)** N stage, and **(F)** pathologic stage.
