# Supplementary material for: m6A-related lncRNAs are potential biomarkers for the prognosis of COAD patients
Source: Front Oncol. 2022 Aug 30;12:920023. doi: 10.3389/fonc.2022.920023 (PMC9472555; doi:10.3389/fonc.2022.920023)
Supplement: Supplementary file 1 [file Table_1.docx]

| **Characteristics** | **Training set (n=214)** | **Validation set (n=212)** | **P value** |
| --- | --- | --- | --- |
| Age |  |  | 0.635 |
| <65 | 80 | 84 |  |
| >=65 | 134 | 128 |  |
| Gender |  |  | 0.917 |
| Male | 114 | 114 |  |
| Female | 100 | 98 |  |
| Stage |  |  | 0.798 |
| Stage I-II | 120 | 118 |  |
| T |  |  | 0.753 |
| T1-T2 | 44 | 41 |  |
| T3-T4 | 170 | 171 |  |
| N |  |  | 0.798 |
| N0 | 126 | 128 |  |
| N1-N2 | 88 | 85 |  |
| M |  |  | 0.482 |
| M0 | 159 | 158 |  |
| M1 | 32 | 26 |  |
