# Supplementary material for: m6A-related lncRNAs are potential biomarkers for the prognosis of COAD patients
Source: Front Oncol. 2022 Aug 30;12:920023. doi: 10.3389/fonc.2022.920023 (PMC9472555; doi:10.3389/fonc.2022.920023)
Supplement: Supplementary file 2 [file Table_2.docx]

| siRNA | Sense (5’-3’) | Antisense (5’-3’) |
| --- | --- | --- |
| siRNA-1 | GCCUUACACAUGUGGGCAUTT | AUGCCCACAUGUGUAAGGCTT |
| siRNA-2 | GCUAUGUGUUCCAAGAUUATT | UAAUCUUGGAACACAUAGCTT |
| siRNA-3 | GCAGAUGCUUCAAUGUAAATT | UUUACAUUGAAGCAUCUGCTT |
| siNC | UUCUUCGAACGUGUCACGUTT | ACGUGACACGUUCGGAGAATT |
| Primer | F primer (5’-3') | R primer (5’-3') |
| UBA6-AS1 | TGACCTGTACTTAGTGCCGAC | AGGCATTTGAACCAGAGTCAC |
| β-actin | CATGTACGTTGCTATCCAGGC | CTCCTTAATGTCACGCACGAT |
