# Supplementary figures and images for: m6A-related lncRNAs are potential biomarkers for the prognosis of COAD patients
Source: Front Oncol. 2022 Aug 30;12:920023. doi: 10.3389/fonc.2022.920023 (PMC9472555; doi:10.3389/fonc.2022.920023)

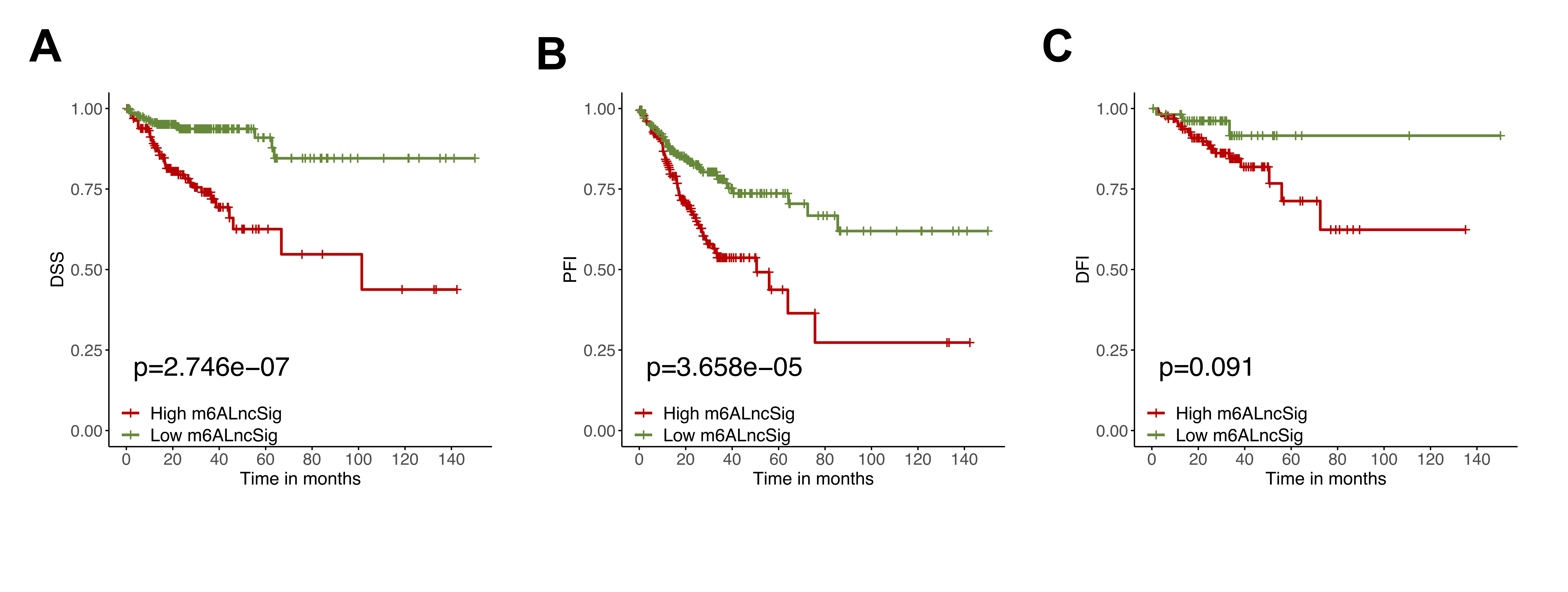

Supplement: Supplementary Figure 1 — (A–C) KM survival curves of DSS, PFI, and DFI between the high- and low-risk groups. [file Image_1.jpeg]

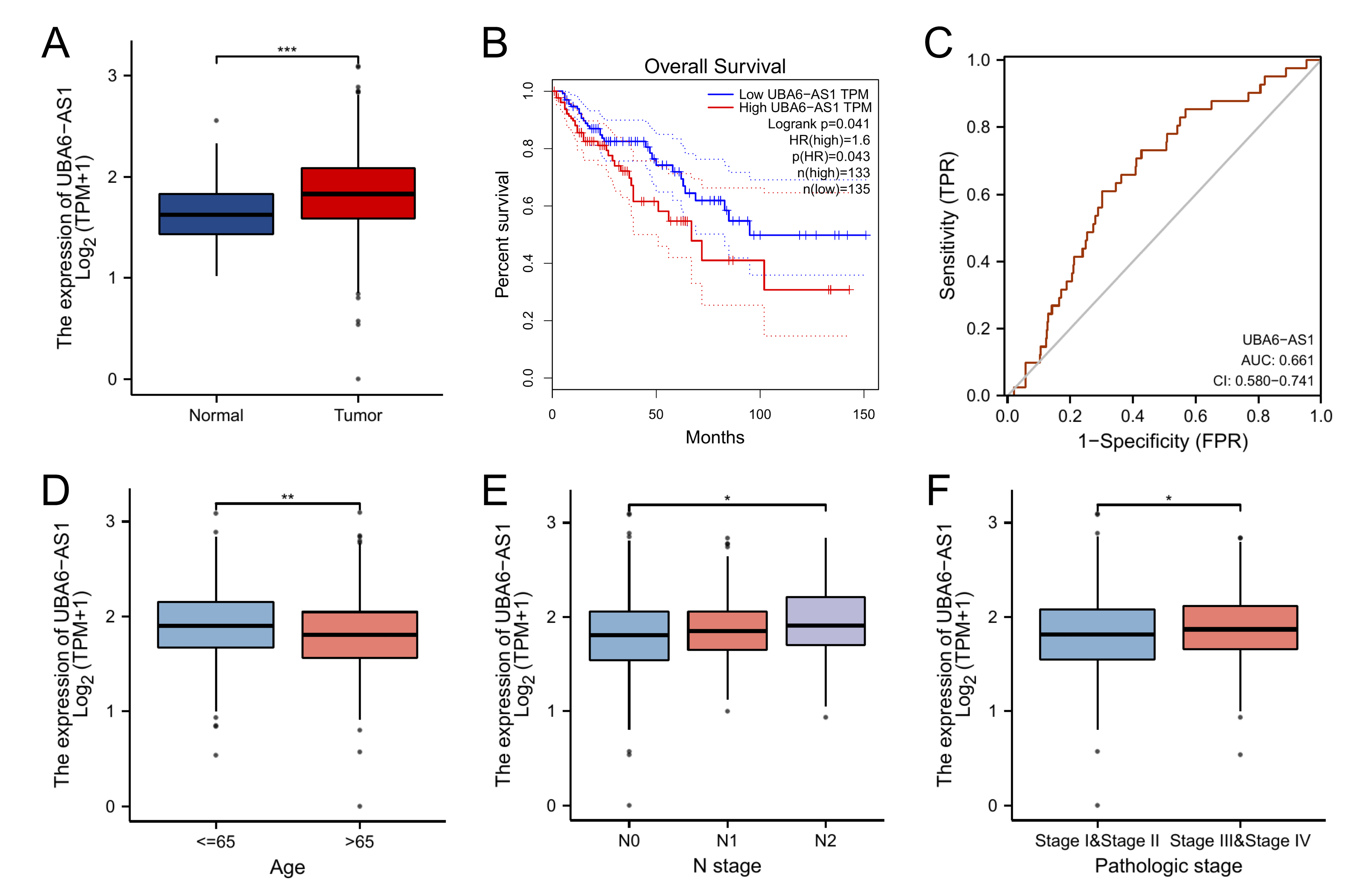

Supplement: Supplementary Figure 2 — The expression and prognosis of UBA6-AS1 in COAD. (A) The differential expression of UBA6-AS1 in COAD cancer and paracancer samples. (B) KM survival curve of UBA6-AS1. (C) ROC curve for UBA6-AS1. (D) Relationship between UBA6-AS1 expression with age, (E) N stage, and (F) pathologic stage. [file Image_2.jpeg]
